# Measurement of length distribution of beta-lactoglobulin fibrils by multiwavelength analytical ultracentrifugation

**DOI:** 10.1007/s00249-020-01421-4

**Published:** 2020-01-31

**Authors:** Maximilian J. Uttinger, Timon R. Heyn, Uwe Jandt, Simon E. Wawra, Bettina Winzer, Julia K. Keppler, Wolfgang Peukert

**Affiliations:** 1 Institute of Particle Technology, Interdisciplinary Center for Functional Particle Systems, Friedrich-Alexander-Universität Erlangen-Nürnberg, Erlangen, Germany; 2 Institute of Human Nutrition and Food Science, Division of Food Technology, Kiel University, 24118 Kiel, Germany; 3 Institute of Bioprocess and Biosystems Engineering, Hamburg University of Technology, Hamburg, Germany; 4 Laboratory of Food Process Engineering, Wageningen University, Bornse Weilanden 9, 6708WG, Wageningen, P.O. Box 17, 6700 AA Wageningen, The Netherlands

**Keywords:** Analytical ultracentrifugation, Sedimentation analysis, Beta-lactoglobulin, Atomic force microscopy, Amyloid fibrils

## Abstract

The whey protein beta-lactoglobulin is the building block of amyloid fibrils which exhibit a great potential in various applications. These include stabilization of gels or emulsions. During biotechnological processing, high shear forces lead to fragmentation of fibrils and therefore to smaller fibril lengths. To provide insight into such processes, pure straight amyloid fibril dispersions (prepared at pH 2) were produced and sheared using the rotor stator setup of an Ultra Turrax. In the first part of this work, the sedimentation properties of fragmented amyloid fibrils sheared at different stress levels were analyzed with mulitwavelength analytical ultracentrifugation (AUC). Sedimentation data analysis was carried out with the boundary condition that fragmented fibrils were of cylindrical shape, for which frictional properties are known. These results were compared with complementary atomic force microscopy (AFM) measurements. We demonstrate how the sedimentation coefficient distribution from AUC experiments is influenced by the underlying length and diameter distribution of amyloid fibrils.

In the second part of this work, we show how to correlate the fibril size reduction kinetics with the applied rotor revolution and the resulting energy density, respectively, using modal values of the sedimentation coefficients obtained from AUC. Remarkably, the determined scaling laws for the size reduction are in agreement with the results for other material systems, such as emulsification processes or the size reduction of graphene oxide sheets.

**Electronic supplementary material:**

The online version of this article (10.1007/s00249-020-01421-4) contains supplementary material, which is available to authorized users.

## Introduction

Amyloid fibrils are found in various fields including medicine, nanotechnology, material science and food science (Knowles and Mezzenga 2016; Cao and Mezzenga 2019). Amyloid aggregates are linked to pathological amyloid fibrils due to their implications in many diseases including Parkinson, Alzheimer and diabetes mellitus (Sipe and Cohen 2000; Chiti and Dobson 2006). The length distribution of amyloid fibrils involved in diseases has a strong impact on their cytotoxicity. Smith et al. (2015) and Tarutani et al. (2016) assume that fragmented amyloid-like aggregated fibrils play a key role in pathogenic seeds for prion-like conversion. One explanation is that amyloid seeds can affect the kinetics of self-templated aggregation (Knowles et al. 2009).

Functional fibril structures are also highly interesting, especially for food applications. The high length-to-height ratio in combination with specific functional groups on the surface makes fibrils from whey proteins an interesting additive for gels, foams and emulsions as stabilizing, emulsifying or gelling agents (Humblet-Hua et al. 2012; Veerman et al. 2003). In particular, relevant effects induced by processing of beta-lactoglobulin (BLG) fibrils and their consecutive fragmentation by the mechanical stressing have been shown before (Oboroceanu et al. 2011; Serfert et al. 2014). Moreover, it is known that fragmentation leads to a slight alteration of the functionality of long fibrils by weakening electrostatic interactions and changing solution properties such as viscosity and effective volume (Koo et al. 2018).

Amyloid fibrils consist of strands up to several micrometers in length with a height of a few nanometers only. These nanofibrils can be formed from a wide variety of proteins and parameters. Common characteristics of amyloid aggregates are the cross-beta structure in which the beta-sheets lie parallel to the fibril axis (Eisenberg and Sawaya 2017; Sunde et al. 1997). In particular, the whey protein BLG undergoes acid hydrolysis prior to the amyloid assembling process; hence, BLG fibrils produced at pH 2 are composed of several peptides (Keppler et al. 2019). Since the conversion rate is rather low, approximately 30–40% of the proteins are converted to fibrils. The residual populations consist of monomers, oligomers and random aggregates and, thus, the samples show a high polydispersity (Heyn et al. 2019).

Adamcik et al. analyzed the structure of amyloid fibril from BLG and in particular their length distributions by atomic force microscopy (AFM) (Adamcik et al. 2010). It was shown that fibrils assemble in multi-stranded helical shape with twisted ribbon-like structure. Moreover, the work revealed a main subpopulation showing heights of around 4.0 ± 0.5 nm and further distinct subpopulations with heights of 2.0 nm ± 0.4 nm 6.0 nm ± 0.4 nm, respectively (Adamcik et al. 2010). However, such statistical screening of microscopic images is very time consuming.

Analytical ultracentrifugation (AUC) is a well established and reliable hydrodynamic method which enables fast and statistically excellent sample characterization and thus gives new insight into fragmentation and assembly during processing. Additionally, AUC measures sample properties directly in solution and therefore excludes drying artifacts including aggregation and other effects that might influence the length distribution of the fibrils.

Fibrils in solution have been characterized via sedimentation analysis using AUC before (Binger et al. 2013; Lee et al. 2009; Pieri et al. 2012). Studies on α-synuclein fibril solutions report an estimate for the molar mass based on the sedimentation properties (Pieri et al. 2012). These fibrils play an important role in neurodegenerative diseases such as Alzheimer (Liu et al. 2002). The precise knowledge of the entire length distribution would help to reveal the underlying formation mechanisms that are essential to better understand the origin of these diseases.

MacRaild et al. (2003) showed the dependence of the sedimentation coefficient as a function of the fibril length for flexible molecules. The degree of flexibility is indicated by extensive tangling and fibril association. On the one hand, this work enables the analysis of heterogeneous mixtures over a broad size range. On the other hand, this approach requires precise knowledge on the structure of the macromolecules as well as an expensive simulation technique to model the flexibility of the molecules upon cross-tangling or entanglement. The precise knowledge of a second parameter such as diffusional or frictional properties is a required prerequisite, which was in this case only feasible through tedious simulations. Moreover, the fibril structure was not well defined due to coiling and entanglement alongside the long axis of the fibrils (MacRaild et al. 2003).

Throughout sedimentation velocity (SV) AUC experiments, diffusion does not superimpose the sedimentation sufficiently in the case of large macromolecules, which is a prerequisite for the precise analysis of length and diameter of such systems. Therefore, it is not possible to obtain information on the molar mass or any additional parameters beyond equivalent size for such systems (Schuck et al. 1998). As demonstrated for the retrieval of the lateral dimensions of graphene oxide sheets or the length distributions for zinc oxide or gold nanorods, it is possible to use sedimentation and optical or frictional properties to gain a thorough insight into distributed properties. It is possible to determine sedimentation properties through AUC experiments, while frictional properties can be calculated from known structures such as cylinders or platelets of the analytes using an expression derived from numerical solutions (Walter et al. 2015; Wawra et al. 2018; Thajudeen et al. 2017a).

The objective of this work is to gain more insight into AUC experiments on BLG fibril solutions. A straightforward approach is developed to study the fibril length distribution from such hydrodynamic experiments. Our samples are produced by stressing of BLG solutions by an Ultra Turrax at defined stress intensity. With this rotor–stator setup, large fibrils are fragmented into polydisperse cylinder-like structures. The formed cylinder-like fibrils are characterized by statistical AFM analysis. Frictional properties of such structures can be calculated by well-known computational methods using numerical solutions for the drag coefficient as a function of the aspect ratio (Hansen 2004).

First, it is shown through well-established simulations of sedimentation data that the remaining monomers and peptides in solution do not affect the sedimentation analysis of the fibril populations. Second, sedimentation coefficients are calculated from statistical AFM analysis, which will be considered a benchmark technique in this work. It is shown that sedimentation properties from AUC and the calculated mean sedimentation properties from AFM statistics agree very well, which in turn validates our AUC results. Next, the sedimentation coefficients and the length distributions obtained from both techniques are compared. The influence of better sample statistics throughout AUC experiments is discussed. Moreover, the effect of an underlying height distribution affecting the results from sedimentation analysis is investigated in detail. Overall, an increased width of the sedimentation coefficient distribution is observed.

In the final part of this work, the shear stress that is applied throughout the Ultra Turrax and the resulting comminution kinetics are analyzed in detail. Our results show that size reduction, hence fibril fragmentation, follows a function of the volume-specific energy input. For this, a power law dependency can be applied to obtain material-dependent parameters that are generally used for the description of comminution processes (Nacken et al. 2017) and further industrially relevant processes (Becker et al. 2001).

In summary, this manuscript outlines how to comprehensively characterize fibril nanorod dispersions. Moreover, the effect of process parameters on fibril formation can now be studied fast and reliably using AUC.

## Theoretical background

### Operational principle of the AUC

The concentration evolution of a sedimenting species in an SV AUC experiment is described by Lamm’s equation:

1 $$\frac{\partial c}{{\partial t}} = \frac{1}{r}\frac{\partial }{\partial r}\left[ {r \cdot D\frac{\partial c}{{\partial r}} - s \cdot \omega^{2} \cdot r^{2} \cdot c} \right].$$

Here, *c* is the concentration of the individual species, *r* is the radial position in the cell, *t* is the time, *s* is the sedimentation and *D* the diffusion coefficient, which both scale with shape and size of the analytes. For species of sufficiently high molecular mass such as fibrils of 31 MDa (length of 500 nm and a diameter of 5 nm), diffusion can be neglected and only sedimentation properties are accessible throughout AUC experiments, which is the case in the presented studies here (Schuck and Rossmanith 2000; Schuck 2000).

In such a case, the sedimentation of macromolecules and nanoparticles can be studied applying gravitational sweep experiments. Throughout gravitational sweep experiments, the attenuation of light is monitored after passing through the centrifugal cell at a fixed radial position *r*_fix_ as a function of time t, angular velocity ω and meniscus position *r*_*m*_. The dynamic change of the rotor speed results in a high resolution of widely distributed sedimentation coefficients which can be measured within one experiment. The sedimentation coefficient in a gravitational sweep experiment is obtained by:

2 $$s = \frac{1}{{\omega^{2} t}} \cdot ln\left( {\frac{{r_{{{\text{fix}}}} }}{{r_{m} }}} \right).$$

### Calculation of sedimentation coefficient for cylinders

The fibrils exhibit a cylindrical shape for which the sedimentation coefficient can be linked to the fibril length and height through frictional properties. The sedimentation coefficient *s* is given as:

3 $$s = \frac{{m_{{F,e{\text{ff}}}} \left( {1 - \frac{{\rho_{S} }}{{\rho_{{F,{\text{eff}}}} }}} \right)}}{f},$$

where *m*_*F*,eff_ is the effective mass of the fibrils, *ρ*_*F*,eff_ is the effective density of the fibrils and *ρ*_*S*_ is the solvent density. The effective properties include a hydration shell of the fibrils; see e.g. Lee und Timasheff 1974 and Walter et al. 2016. The frictional coefficient is denoted as *f*. Assuming a cylindrical shape with cylinder length l, height h and the aspect ratio *q* with *q* = *l*/*h*, Eq. (3) can be rearranged to (Walter et al. 2015):

4 $$\frac{1}{s}\left( {\frac{{3h^{3} }}{{2q^{2} }}} \right)^{\frac{2}{3}} \left( {\rho_{F,eff} - \rho_{S} } \right) - 18\eta \frac{f}{{f_{0} }} = 0.$$

**Fig. 1:**
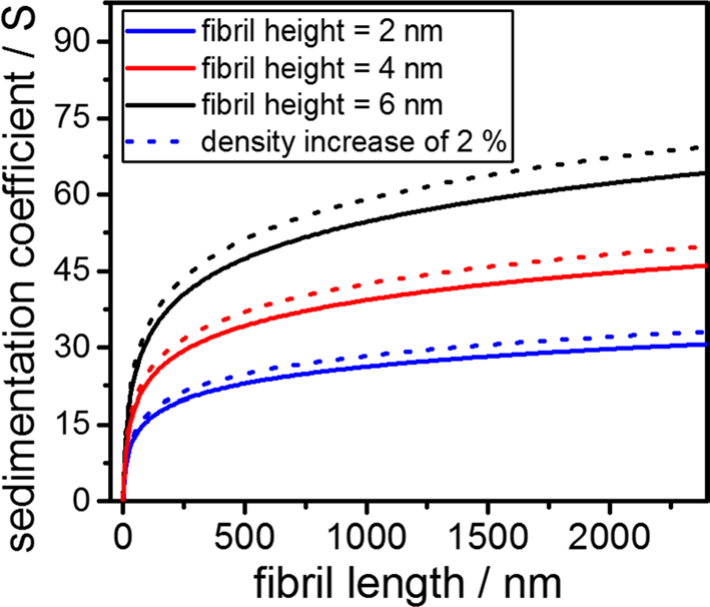
Theoretical dependency of the sedimentation coefficient on the fibril length for different diameters. The fibril density is assumed to be the density of monomeric BLG. Further, the effect of a densification of 2% is shown as well

The solvent viscosity is denoted as *η*. The frictional ratio *f*/*f*_0_ relates the frictional coefficient of the particle under investigation to the frictional coefficient *f*_0_ of an equivalent sphere with the same volume. The translational friction coefficients for cylinders for different aspect ratios were investigated by Steen Hansen. It was shown that *f*/*f*_0_ can be expressed as a function of the logarithm of the aspect ratio, according to Hansen (2004):

5 $$ \begin{aligned}\frac{f}{{f_{0} }} &= 1.0304 + 0.0193{\text{k}} + 0.06229{\text{k}}^{2} + 0.00476{\text{k}}^{3} \\&\quad+ 0.00166{\text{k}}^{4} + 2.66\, \times \,10^{ - 6} {\text{k}}^{7}\end{aligned} $$

with *k* = ln(*q*). Hence, the sedimentation coefficient s can be calculated via Eqs. (4) and (5) as a function of the cylinder length for known density and diameter of the cylinder. Figure 1 shows the sedimentation coefficients for three different diameters assuming the fibril density to be equal to 1331.6 kg m^−3^, which is the inverse of the partial specific volume of BLG. Moreover, the calculations were repeated for the same range of fibril lengths, but with an increased density of 2% with respect to the density of native BLG. As will be shown in a later part of this manuscript, the fibrils show an increase in density of 2% in comparison to their building blocks. The resulting relationship between sedimentation coefficient and fibril length for an increased density is further depicted in Fig. 1.

## Materials and methods

### Assessment of effective fibril densities using molecular dynamics

One important prerequisite for the precise evaluation of the sedimentation properties of amyloid fibrils is the knowledge of the effective density. For a detailed analysis, molecular dynamics (MD) simulations of amyloid fibrils in suspension were conducted to assess the increase of effective density of densely packed beta-sheet amyloid-like structures with respect to native BLG.

All MD simulations and analysis were performed using the GROMACS software package version 5.1.1,(Berendsen et al. 1995; van der Spoel et al. 2005; Lindahl et al. 2001) and the OPLS-AA (all-atom) force field (Kaminski et al. 2001). The visual molecular dynamics (VMD) program was used for the visualizations (Humphrey et al. 1996). The simulated protein structures for BLG have been obtained from PDB 3PH5 and a model for typical beta-sheet amyloid-like structures was obtained from PDB 2MXU. A corresponding amyloid structure from BLG has not yet been determined to the best of our knowledge. More details of the MD algorithm are provided in the electronic supporting information (ESI).

Calculations of the effective density of native BLG and aggregates from MD simulations involves an approximation of the volume fraction, which is based on the part of the protein that is analyzed and the number of solvent molecules inside the protein structure. Assuming a hydration layer of approximately 3 Å thickness drastically overestimates the covered volume, overall leading to a severe underestimation of the density (for native BLG: approximately 1190 kg m^−3^ simulated vs. 1331.6 kg m^−3^ experimental), which is similar to earlier findings by others; see in the publications Andersson and Hovmöller (1998) and Quillin and Matthews (2000). Similar to the work from Quillien et al. (Quillin and Matthews 2000), no explicit hydration layer was therefore assumed in our simulation. Instead, only tightly bound water molecules were considered along with their covered volume. Therefore, a discrete voxelmap represented the volume covered by the pure protein structure, with every protein atom represented by its VdW volume. The resolution of the voxelmap was determined to be 0.05 nm to yield convergent results (data not shown). The herewith covered volume included very few solvent molecules. Additional solvent molecules were considered to be tightly bound if they were surrounded by an additional volume p of protein within a distance of 3 A. Finally, completely closed cavities, i.e*.*, regions without contact to the bulk solution, were detected and hence added to the protein volume. All molecules within the obtained volume contributed to the effective mass and density. The exact value of the additional volume *p* was expected to be between 30 vol.% and 60 vol.%, defining solvent regions to be “tightly bound”, if protein surrounds roughly half of the nearby volume. The value of *p* in our case was determined using a model-based fit of the data based on the experimental value of native BLG. Our results showed a value of *p* = 38 vol.% and thus a simulated effective density of 1330.27 kg m^−3^, which deviates less than 0.1% from the experimental value.

The settings remained similar for the fibril analysis to allow for a relative comparison. Notably, without further validation (i.e, a priori determination and validation of *p*), this approach does not necessarily yield absolute effective density values. However, its main objective in this work is the thorough determination of relative density changes of amyloid aggregates and their monomeric building blocks. The precise knowledge of relative density changes enables comprehensive data analysis of AUC and auxiliary AFM measurements.

### Fibril preparation

Functional amyloid fibril solutions were prepared according to a protocol of Keppler et al. (2019). A 2.5 wt% BLG (Davisco Foods International, Inc. Eden Prairie, USA) solution was prepared and set to a pH of 2.0 by adding 6 M HCl. 150 mL of the protein solution was filled into 250 mL Schott glasses and heated in a water bath to 90° C for 5 h under continuous stirring at 350 RPM using a magnetic stirrer (2mag MIXdrive 6HT, 2mag AG, Munich, Germany*)*. For dilution of the fibril solutions, water at pH 2 was used.

### Stressing of fibrils using Ultra Turrax

Fibril solutions were subjected to stressing using a T 25 Ultra Turrax with an S 25 N–25 F dispersion tool (IKA®—Werke GmbH and CO.KG, Staufen, Germany). 100 mL of the solution was stressed for *t*_stress_ = 5 s, 30 s, 60 s and 90 s at a rotational speed *ω*_stress_ of 11, 13, 16, 19, 22 and 24 krpm. The concentration while stressing was 10 g L^−1^. A measure of the acting stress is the stress intensity (SI), i.e. the energy input per mass, which is a well-established quantity for comminution experiments (Damm et al. 2015; Knieke et al. 2010,2011). A measure for the acting stress intensity of an Ultra Turrax experiment is in first approximation provided by Eq. (6) which is simply the number of rotations of the Ultra Turrax throughout the course of one experiment:

6 $$n = \frac{{\omega_{{{\text{stress}}}} \cdot t_{{{\text{stress}}}} }}{2\pi }.$$

The rotational speed is denoted as *ω*_stress_ and the stressing time as *t*_stress_. Upon stressing of the fibril solutions, the temperature was controlled. The pH of the solution was checked before and after stressing to exclude pH-induced effects. To minimize effects due to aggregation, the samples were measured directly after stressing. A list of all samples alongside the rotational speeds and stressing times is provided in the ESI.

### Scaling law for size reduction

In size reduction by grinding, emulsification or spraying, the mean particle size scales with the applied energy per volume (Zhang et al. 2012; Becker et al. 2001):

7 $$x_{{{\text{mean}}}} = a \cdot E_{V}^{ - b}$$

with the constants *a* and *b*. Typically, parameter *b* varies between 0.3 and 0.8 depending on the applied type of stress and the material. The volume-specific energy input *E*_*V*_ is obtained from the acting power *P* over time *t* in the volume *V* (Zlokarnik 1999):

8 $$E_{V} = \frac{P \cdot t}{V}.$$

Here, the fibrils are stressed in an Ultra Turrax where the applied stress is a function of the rotor revolution *n*. In rotating and stirred systems, the dimensionless Newton number Ne is a measure for the power *P* and can therefore be used to describe processing via Ultra Turrax:

9 $${\text{Ne}} = \frac{P}{{n^{3} d_{R}^{5} \cdot \rho_{S} }} = f\left( {{\text{Re}}} \right),$$

with the rotor diameter denoted as *d*_*R*_. The Newton number is a direct function of the Reynolds number Re:

10 $${\text{Re}} = \frac{{n \cdot d_{R}^{2} \cdot \rho_{S} }}{\eta }.$$

For Reynolds numbers above 10^4^ in the turbulent regime, the Newton number remains constant and is merely a function of the stirrer and slit geometry (Håkansson 2018).

## Atomic force microscopy AFM

### Sample preparation

Prior to the AFM measurements, samples were diluted to 0.1 mg mL^−1^ to optimize the concentration for coating the samples onto a substrate. Next, 2 µL of the samples were drop-coated onto a mica substrate and washed with 2 × 200 µL pure water to minimize sample aggregation upon drying onto the substrate.

### Measurement

The dimensions of the individual fibrils were measured with an AFM (nanoscope III; digital instruments). The resolution was set to 512 pixels × 512 pixels for a 5 × 5 μm measuring surface (∼9.77 nm/pixel). Imaging was performed in tapping mode with an NSC15/AlBS probe (frequency 325 kHz, spring constant 46 N m^-1^ and nominal tip radius < 10 nm) applying a scan rate of ≤ 0.7 Hz. A total area of 25 μm^2^ was measured.

### Image analysis

AFM data were transformed to pictures and imported to AxioVision 4.8 (Carl Zeiss Imaging Systems, Germany) where evaluation was performed in several steps. After contrast imaging, a conversion of pixel to nm scale was achieved by taking the total length from the AFM image scan field as reference. Finally, fibril lengths were detected and cross-checked to exclude non-separated and stacked fibrils induced by drying on the coated mica glimmer. All fibrils were counted and the length of each individual fibril was obtained. The minimum length used for image analysis was ∼50 nm. If fibril lengths extended over the edge of the substrate, these were excluded from the analysis.

## Analytical ultracentrifugation

### Simulation of AUC data

Sedimentation data as retrieved from AUC experiments were simulated to check whether or not small species in solution influence the analysis of the fibril populations (Patel et al. 2018). Gravitational sweep experiments were simulated via a well-established brownian dynamics (BD) algorithm. Details of the algorithm have been described elsewhere (Thajudeen et al. 2017b; Díez et al. 2011). The meniscus of the solution was set to 5.9 cm and the bottom of the cell to 7.2 cm from the rotor axis. The detector was positioned at a radial distance of 6.9 cm. To study the influence of the non-aggregated material (NAM) in the fibril solution throughout the AUC experiments, three simultaneously sedimenting species were simulated. One species was assigned the monomer parameters, namely a sedimentation coefficient of 4 s and a frictional ratio of *f*/*f*_0_ = 1.26. The effective density of the monomers was set to 1331.6 kg m^−3^. The two other species were assigned the parameters of representative fibril-like species with a sedimentation coefficient of 50 S and 100 S and an effective density which is 2% higher than the monomer density. The assumption of a certain density increase of fibrils with respect to the native protein structure arises from the expectation that densely packed beta-sheet structures should contain fewer voids and water-filled pockets than the native protein. The increase of 2% has been further estimated using structural investigations via MD simulations (see “MD simulations of the densification of fibril-like structures”). The frictional ratio of the fibril-like species was set to 1.5. The simulated experimental time was 7200 s at 30,000 rpm which resulted in complete sedimentation of the fibril species.

### Gravitational sweep experiments

A modified preparative centrifuge, type Optima L-90 K from Beckman Coulter, with an integrated UV/visible multiwavelength detector was used for the rotor speed gradient experiments. The rotor speed was held constant and set to 30,000 rpm to allow for accumulations of the light intensity on the UV/visible detector. Sedimentation data were acquired for wavelengths between 220 and 450 nm. The buffer solution was taken as a reference when converting intensity data to absorbance. The temperature was set to 20 °C throughout all measurements. Titanium centerpieces with an optical path length of 12 mm were used for all experiments. Further details of the applied multiwavelength optics as well as the data evaluation can be found in literature (Walter and Peukert 2016; Walter et al. 2014). Prior to the measurements, the samples were diluted to 1 g L^−1^. Afterwards, the protein concentration was controlled by measuring the extinction of the samples at 280 nm and 290 nm. The optical density was in the order of unity at a wavelength of 280 nm for each fibril solution under investigation.

## Results and discussion

### Molecular dynamics simulations of the densification of fibril-like structures

For the detailed analysis of sedimentation properties of cylinder-like fragmented fibrils, the precise knowledge of the effective density is crucial. For this, we use the inverse of the partial specific volume as a first approximation for the density of native BLG (Brown und Schuck 2006; Harding et al. 2015).

However, as structural alterations upon fibril formation induce changes in the effective density, MD simulations were carried out to investigate such changes in more detail. As a first validation step, native BLG is simulated and results are compared with the inverse of the partial specific volume. Next, fibril structures were analyzed and density changes were retrieved from the MD simulations.

**Fig. 2:**
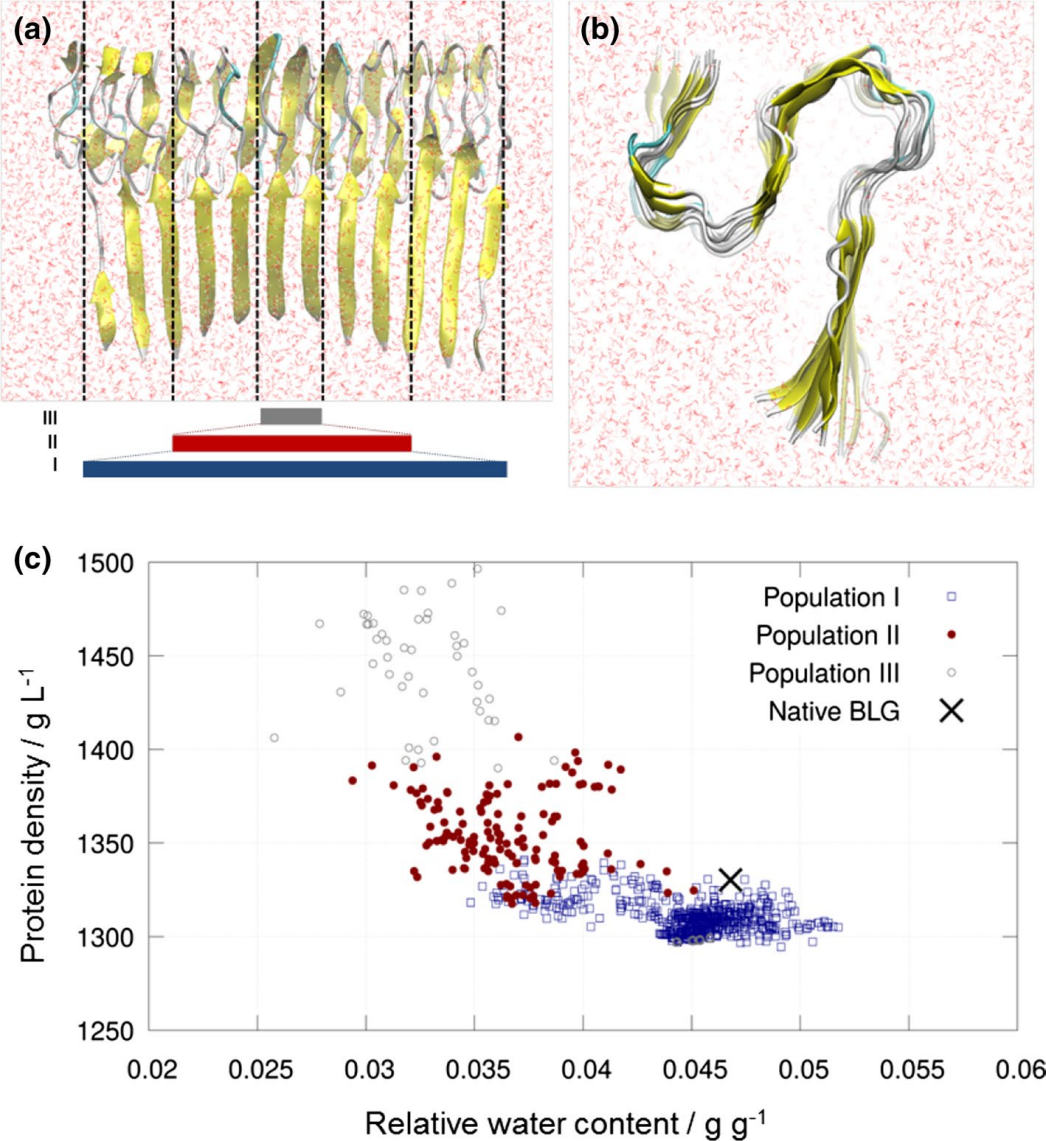
MD simulations of fibrils and native BLG to determine the presumed increase of protein density of fibrils vs. native BLG. Visualization of fibrils along the long (**a**) and cross-sectional (**b**) axes, with solvent molecules indicated in red. Densely packed fibrils with infinite length can be mimicked by considering a limited volume in the center of the simulation domain (marked by red bar, Population II). The exact position of the considered volume is defined by offset and width. In the cross-sectional view, the lack of solvent molecules within the densely packed fibril structure is visually evident. Three different series of measurements have been performed, indicated by the colored bars in (**a**): **I** for the whole simulated fibril, containing 8–12 fibril folds and thus also including its partially unfolded ends; **II** for the densely packed center of the simulated fibril, containing 4–6 folds; **III** for the extremely densely packed inner part (central two folds) of the structure, which have not been considered for analysis alone, since accuracy of such small structural parts is expected to be too limited. **c** Plot of determined densities vs. relative content of water molecules, separated for the three sub-analyses and for natively folded BLG (black cross)

MD simulations were performed as described in the methods section. The increase of protein density and, at the same time, decrease of incorporated solvent (mostly water) molecules within the densely packed beta-fibril structure is clearly evident (Fig. 2a, b). Thus, a density of 1330 kg m^3^ with a water content of 0.047 g g^−1^ was determined for natively folded BLG using density measurements as described in the methods section (black cross in Fig. 2c).

Applying the same simulation setup, regions of differently packed structures, hence protein densities and the corresponding water contents, were identified for limited simulated fibril structure sizes. As expected, the fibril structures are loosely packed at the open ends of the 12-fold fibrils, hence the density is lower along them. This observation is indicated by “Population I” in Fig. 2 a and c. The overall protein density for this region was determined as (1310 ± 8) kg m^−3^ with a relative water content of (0.045 ± 0.003) g g^−1^.

In comparison, the protein density increased to (1354 ± 21) kg m^−3^ and the relative water content to (0.037 ± 0.003) g g^−1^ for “Population II”, representing the central, more densely packed region consisting of four to six folds. The most central pair of folds (“Population III”) yields extremely high effective densities with an average of more than 1400 kg m^−3^. However, it is presumed that the limited available data in this region are too scarce for useful comparison.

Overall, the protein density of the packed fibril region (Population II) is considerably higher compared to native BLG, namely (+ 1.7 ± 1.5%), and also compared to loosely packed fibrils that is partially unfolded, as represented by Population I, namely (+ 3.3 ± 1.7)%. This clearly shows an increase of protein density of the packed fibril structures in the order of magnitude of 2% with respect to native BLG. However, the upper limit of the density increase cannot be accurately determined and an increase of more than 2% is unlikely.

### Statistical AFM image analysis

**Fig. 3:**
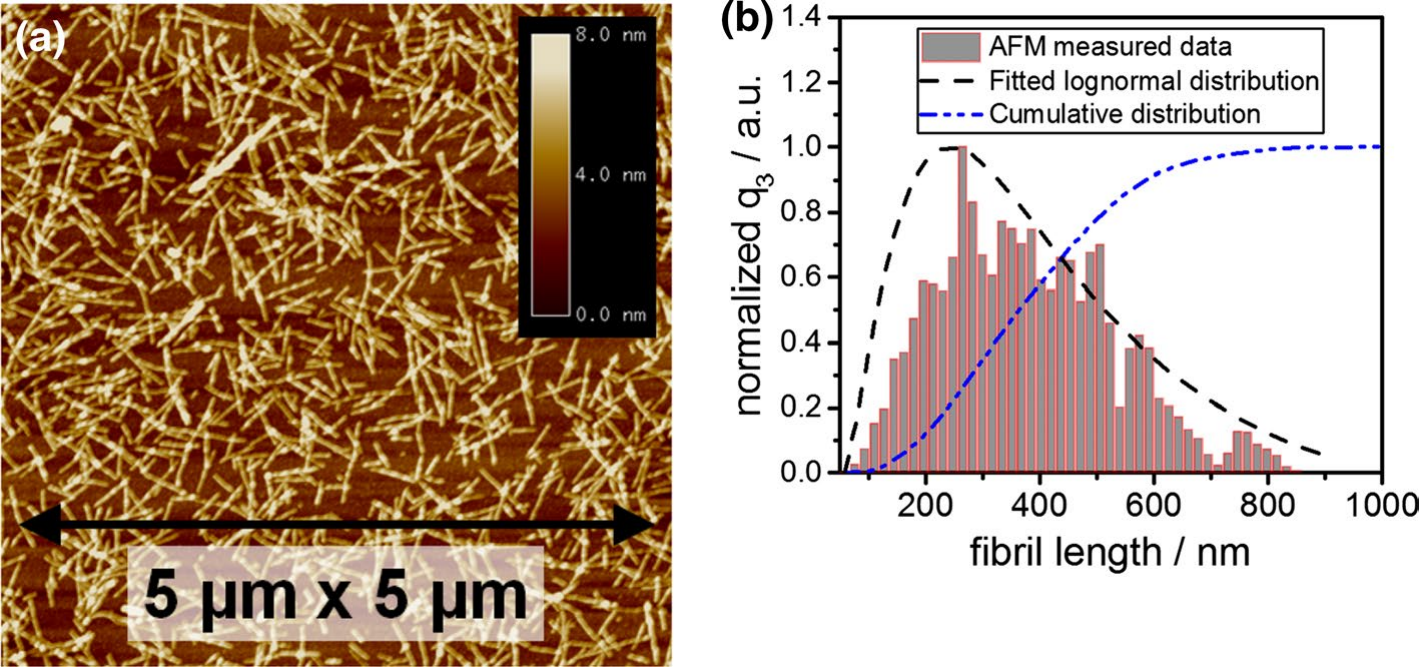
**a** AFM image of stressed BLG fibrils deposited onto mica via drop coating. The dimensions of the waver are 5 µm in each direction. **b** Normalized mass density and mass cumulative distribution of the fibril length from analyzing the AFM image. Mass density distribution is fitted to perform further analysis steps. The stressing conditions in the Ultra Turaxx were 19 krpm for 60 s

As outlined in the introduction to this manuscript, the fibril dispersions were shear stressed in an Ultra Turrax at predefined rotor revolutions to induce fragmentation and to obtain cylindrical fibrils. For each sample, AFM image analysis was carried out to verify whether or not the preprocessing step was successful. One representative scan of the sample which was stressed at 19,000 rpm for 60 s is shown in Fig. 3a. Clearly, the deposited fibrils are of cylindrical shape. It is further evident that the fibrils are well defined and can be measured independently from their neighbors. Fibril entanglement and fibril bending are also not observed. Hence, the preprocessing step is considered successful. Fragmentation due to stressing of the fibrils is further evident when comparing our results from Fig. 3a with the AFM measurements of equivalent fibril solutions that were not subject to stressing as shown by Heyn et al. (2019).

The statistical AFM image analysis was conducted by counting over 2000 particles for each sample. Therefore, the number of AFM images was chosen such that reliable statistics on the overall fibril length range was achieved for each individual sample. The number- and mass-weighted fibril length density distributions were obtained via a classification algorithm. The equation for the calculation of mass and number densities is provided in the ESI. The fibril length distributions are fitted with a lognormal distribution. One representative mass density distribution is shown in Fig. 3b. This particular sample has a mean fibril length of 395 nm and a minimum and maximum fibril length at 80 nm and 810 nm, respectively. The statistical AFM analysis and the retrieved fibril length distributions of further samples are shown in the ESI.

### BD simulations influence of remaining non-aggregated material in solution

In this section of the manuscript, the difference in the sedimentation properties of NAM and fibrils are investigated theoretically. The simulated data clearly indicate that the sedimentation properties of the fibrils are not affected by the presence of NAM. The conversion from native BLG and peptides to amyloid fibrils amounts up to 45%, as shown by filtration experiments (Heyn et al. 2019). The sedimentation properties of NAM differ significantly from those of amyloid fibrils. The selection of the experimental parameters for the AUC experiments targets the detection of the fibrils in dispersion. However, it is not possible to detect NAM in the same experiment, since it has not yet passed the detector position. Consequently, NAM does not sediment from meniscus to bottom within the experiment and thus remains in solution. Slight sedimentation of NAM might result in small intensity fluctuations at the detector and will further influence the absolute dilution of the larger sedimenting species. This would directly translate into an error in the sedimentation coefficient distribution. Details can be taken from respective publications, e.g., see the work from Scott and co-workers (Patel et al. 2018). To evaluate the influence of the presence of NAM on the apparent sedimentation properties of fibrils in the AUC experiment, auxiliary BD simulations were carried out. Therefore, three representative species were considered. Two represent BLG fibril-like macromolecules which sediment at 50 S and 100 S, which are the properties of fibrils which were found experimentally. A sedimentation coefficient of 4 S is assigned to the remaining species which is NAM.

**Fig. 4:**
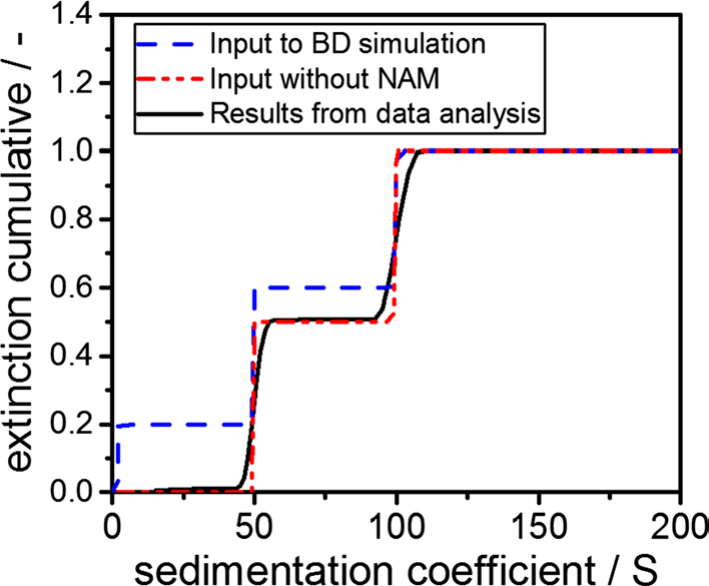
Cumulative sedimentation coefficient distributions as input to BD simulations (blue dashed line) alongside the distribution without the monomer and a cutoff at 10 S (red dashed–dotted line). Furthermore, the analyzed distribution from data analysis is depicted. The input to the BD simulation and the result from analysis of simulated data matches perfectly

The overall results of the BD simulations are summarized in Fig. 4. First, it shows the input distribution to the BD simulation as a blue dashed line. Second, since the NAM will not be obtained by the analysis, it is excluded from the input distribution and the remaining and renormalized distribution is shown as a red dashed line in Fig. 4. For this, a cutoff at 10 S was introduced. Finally, the result from the analysis is shown in Fig. 4 as a black straight line. Evidently, the renormalized input distribution and the result form the analysis match perfectly. These results indicate that any interference caused by NAM is absent and the sedimentation properties of fibrils can be analyzed accurately regardless of the presence of NAM. This enables a comprehensive AUC analysis of cylinder-like fibrils in solution.

### Sedimentation properties of fragmented cylinder-like fibrils

**Fig. 5:**
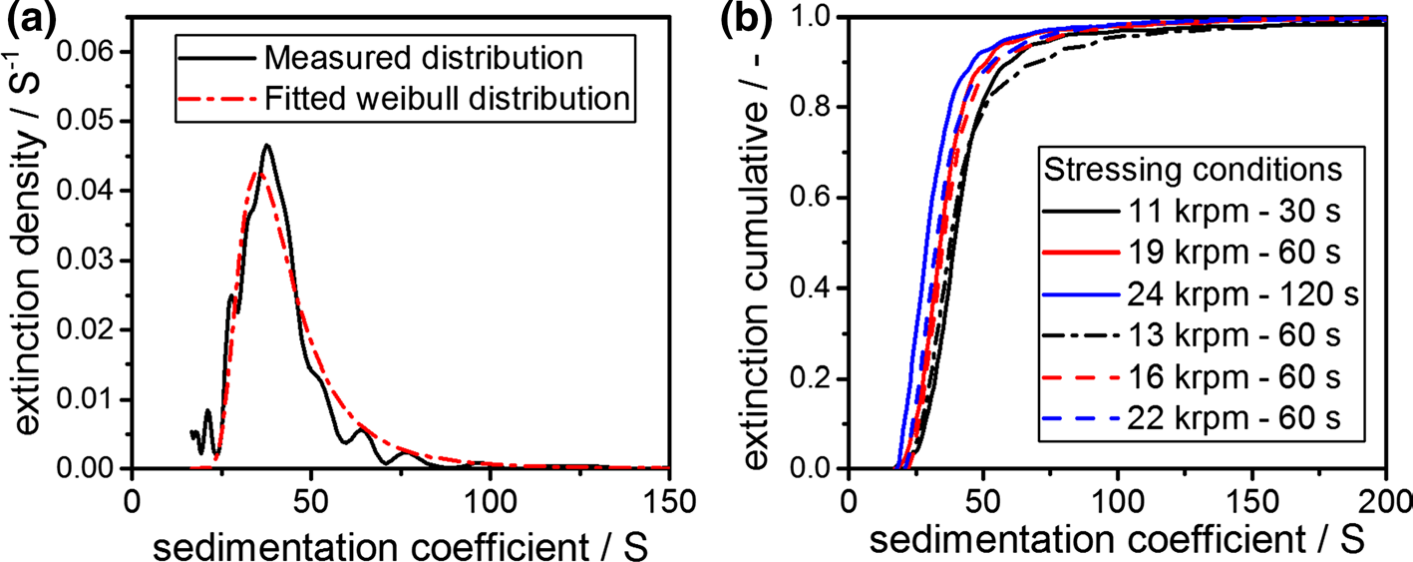
**a** Exemplary measured sedimentation coefficient density distribution from AUC and applied Weibull fit for a stressed (19,000 rpm for 60 s) sample using the rotor stator setup. **b** Extinction cumulative sedimentation coefficient distributions as measured via AUC experiments for different stressing conditions. The sedimentation coefficient distributions shift to lower values with increasing stress intensity. The stressing time as well as the rotational speed of the Ultra Turrax was varied to compare the results from various stress intensities

Stressed samples were diluted to 1 g L^−1^ to optimize the optical signal throughout the AUC experiments. An exemplary result from a single AUC experiment is provided in Fig. 5a. Since the retrieved distribution is tailing, it resembles a Weibull-type distribution and was therefore fitted accordingly. From integration of the measured distribution, the cumulative sedimentation coefficient distributions are obtained. Figure 5b depicts six representative cumulative sedimentation coefficient distributions from samples that were stressed at different stress levels. Evidently, the distributions are continuous and shift as a function of the stress intensity.

A comparison with the theoretical relationship between sedimentation coefficient and fibril length indicates that the measured mean fibril lengths are in the order of 300–600 nm, which is in line with our AFM measurements. A detailed discussion of the retrieval of mean fibril length as well as the entire fibril lengths distribution from AUC experiments is presented in later parts of this manuscript. However, it is already evident in Fig. 5b that all sedimentation coefficient distributions have their minimum at a defined sedimentation coefficient. This observation points toward the fact that the minimal fibril length is almost identical, independent of the applied energy input.

To calculate the minimal length, a thorough understanding of the hydrodynamic properties of fibrils is developed first. Therefore, mass density distributions from AFM measurements were converted to sedimentation coefficient distributions assuming a constant fibril height of 4.7 nm. This value corresponds to the mean fibril heights from literature of 4.0 nm plus the height of two surrounding water layers that are attached to the surface of the fibrils. We verified these values by measuring the height of the samples using AFM on 12 different fibrils. Our results confirmed a height of 4 nm (3.8 ± 0.5 nm). A detailed analysis of the fibril height via statistical AFM analysis has been conducted in literature (Adamcik et al. 2010) and showed similar results. The effective density of the fibril structures in solution were taken from our MD simulations. The frictional ratio *f*/*f*_0_ of the fibrils in solution was calculated based on Eq. (5).

**Fig. 6:**
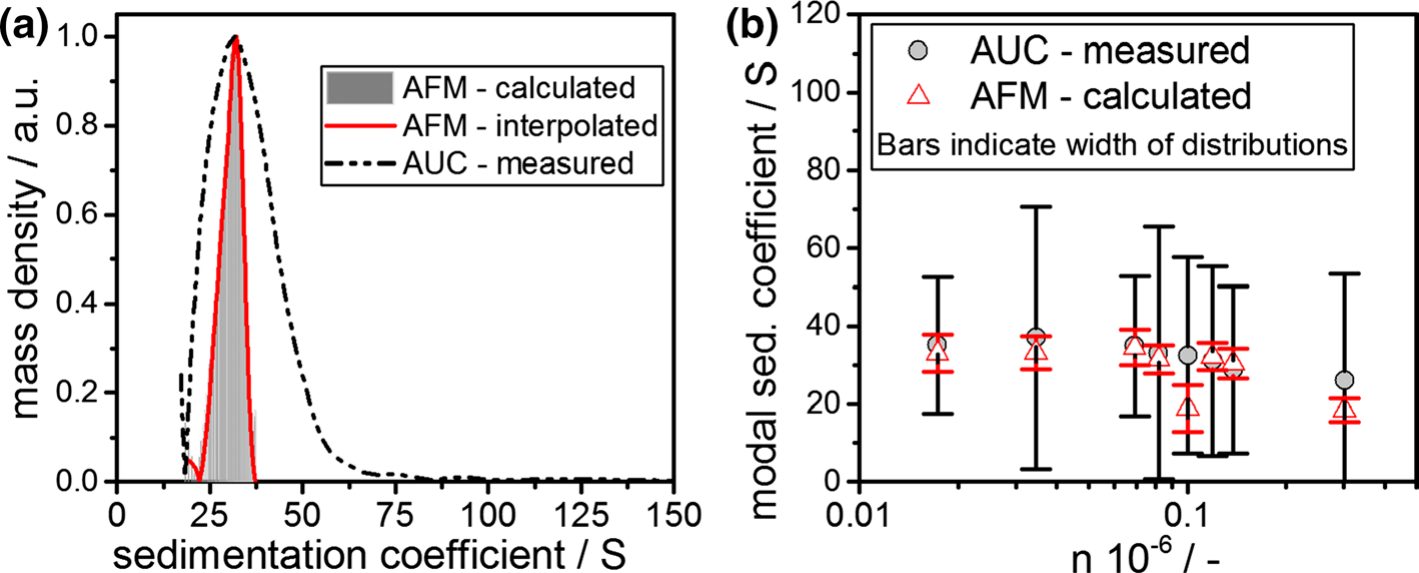
**a** Mass density sedimentation coefficient distribution as measured from AUC (black dashed line) alongside the sedimentation coefficient distribution as calculated from AFM analysis for an assumed fibril height of 4.7 nm for different stressing conditions. The AFM distribution is interpolated for further data analysis. **b** Modal mass-weighted sedimentation coefficients from AUC experiments and from modal mass-weighted AFM analysis for different stressing conditions. The width of the analyzed distribution is indicated as bars

The calculated sedimentation coefficient distribution from a representative AFM measurement alongside the measured distribution from AUC is depicted in Fig. 6a. The density distributions were therefore converted from extinction weighted to mass weighted to enable a thorough comparison with AFM data. This conversion was carried out under the assumption that each individual fibril consists of individual subunits, each contributing equally to the extinction signal. Finally, our results show that the modal sedimentation coefficients from AUC and AFM are identical. This in turn proves the correctness of effective density which was retrieved from the MD simulations and the approach taken in this work.

All samples were analyzed accordingly and the sedimentation coefficient distributions from both, AUC and statistical AFM analysis were compared for the applied rotor revolutions *n*, which is a measure of the volume specific energy input. Here, the retrieved results are summarized in Fig. 6b. In this plot, the data points indicate the modal sedimentation coefficients of the underlying distributions. Moreover, the bars represent the width of each individual distribution. Notably, each sample was monomodal. The modal sedimentation coefficients match for all stress intensities. The relative differences are in the order of 2–3%. Also, with increasing rotor revolution and thus stress intensity, the modal sedimentation coefficient decreases from 42 to 25 S. This is evidence for an underlying comminution kinetic. Conclusively, mean sedimentation properties of cylinder-like fibrils measured directly in dispersion can be predicted when knowing the structure and the effective density. In summary, this enables a comprehensive, fast and efficient analysis of the mean sedimentation properties of fragmented cylinder-like fibrils via AUC in dependence of the applied process parameters.

However, the width of the sedimentation coefficient distributions from both techniques differs considerably. While the AUC results exhibit a width of ± 20 to ± 40 S, statistical AFM analysis yields a much smaller width of ± 5 S. This distinct difference is in parts due to better sample statistics of the AUC and clearly highlights the strength of fast AUC analysis over tedious AFM counting. The increase of the broadening can further be attributed to the fact that the height of the fibrils is not constant and therefore alters the sedimentation coefficient as can be seen from Eq. (4). Since this is essential to the understanding of fibrils, the influence of a two-dimensional height and length distribution on sedimentation properties will be discussed in subsequent chapters of this manuscript.

### Retrieval of the length distribution from AUC experiments and AFM image analysis

In literature, it is discussed how the length distribution of fibrils alongside their number concentration are involved in amyloid diseases and therefore have a significant impact on their cytotoxicity; see e.g. Smith et al. (2015). Hence, fast and reliable methods that determine the entire fibril length distribution efficiently are needed. We converted the sedimentation coefficient distribution measured by AUC to fibril length distributions applying Eq. (4) and assuming an effective height of 4.7 nm. Again, the effective fibril density was retrieved from MD simulations.

**Fig. 7:**
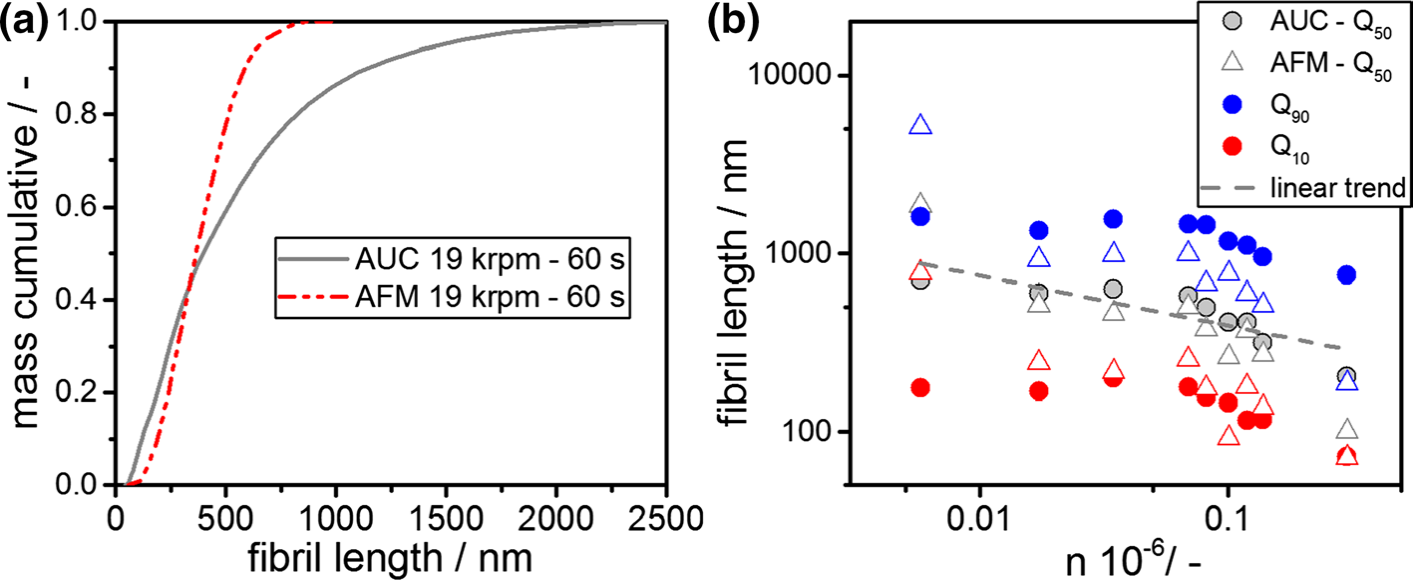
**a** Mass cumulative fibril length distribution as calculated from AUC measurements taking into account the entire sedimentation coefficient distribution and an assumed fibril height of 4.7 nm (gray straight line) alongside the measured length distribution from AFM measurements (red dashed line). **b** Fibril length at different fractions of the mass cumulative distribution (Q_10_, Q_50_ and Q_90_, indicated by different colors, respectively) from AFM measurements (triangles) and AUC measurements (circles) as a function of different stressing conditions. The overall trend is indicated with a black dash–dotted line

Figure 7a shows a representative distribution for a sample that was stressed for 60 s at 19,000 rpm. The increased width of the sedimentation coefficient distributions from AUC directly translates into an increased width of the length distribution. Nevertheless, as can be seen in Fig. 7a, the distributions agree well in the lower part of the distribution, hence for fibril lengths below 700 nm. Furthermore, the mean fibril lengths match.

Consequently, the fibril lengths were retrieved for all samples from AUC measurements and compared with the results from statistical AFM measurements. The results are summarized in Fig. 7b. The mass cumulative fractions of 0.1 and 0.9 alongside the mean fibril length, denoted as Q_10_, Q_90_ and Q_50_, respectively, are displayed as retrieved from both analytical techniques.

Analogous to the discussion of sedimentation properties, the mean fibril length is reproduced by both techniques and the trends match quite well. The underlying comminution kinetic is evident. It is interesting to note that the fibril lengths at a mass cumulative fraction of 0.1 match to a reasonable degree. At higher fibril lengths, represented by mass cumulative fractions of 0.9 in Fig. 7b, these approximations do not hold any more. Large fibril fragments are expected to be more flexible throughout the AUC experiment as concluded from the high ratio of contour to persistence length. Moreover, the AFM measurement is carried out at dried state where drying effects may lead to a more stiff conformation and hence reduced flexibility (Adamcik and Mezzenga 2012).

In summary, these results demonstrate the possibility to determine mean fibril lengths and fibril length distributions up to a length of around 700 nm. Generally, the upper parts of the retrieved length distributions are not accurately described. If the ratio of contour to persistence length exceeds a certain value, the approximation of a cylinder-like structure breaks down because of increased flexibility of the fibril molecules. In such cases, only sedimentation properties should be used to quantify and describe fibril properties directly in solution. Notably, it is possible to investigate fibril structures of irregular shape. However, one prerequisite is the precise knowledge of the frictional properties of these structures. For known structures, well-validated strategies exist to calculate frictional properties from a structure, such as well-known bead modelling tools (García de la Torre and Rodes 1983).

### Detailed analysis of the sedimentation coefficient distribution based on a two-dimensional height and length distribution

As mentioned in the introduction to this manuscript, fibril structures do not exhibit a constant height. Literature studies demonstrate that there are defined subpopulations of various fibril heights: one main subpopulation at 4.0 ± 0.5 nm and two further distinct subpopulations at 2.0 nm ± 0.4 nm and 6.0 nm ± 0.4 nm, respectively (Adamcik et al. 2010). Therefore, we investigated the impact of a 2D distribution in length and diameter of the fragmented and cylinder-like fibrils on the sedimentation coefficient distributions. It will be shown how the increased width of the distributions from AUC measurement can partly be attributed to the various heights of fibrils in solution.

**Fig. 8:**
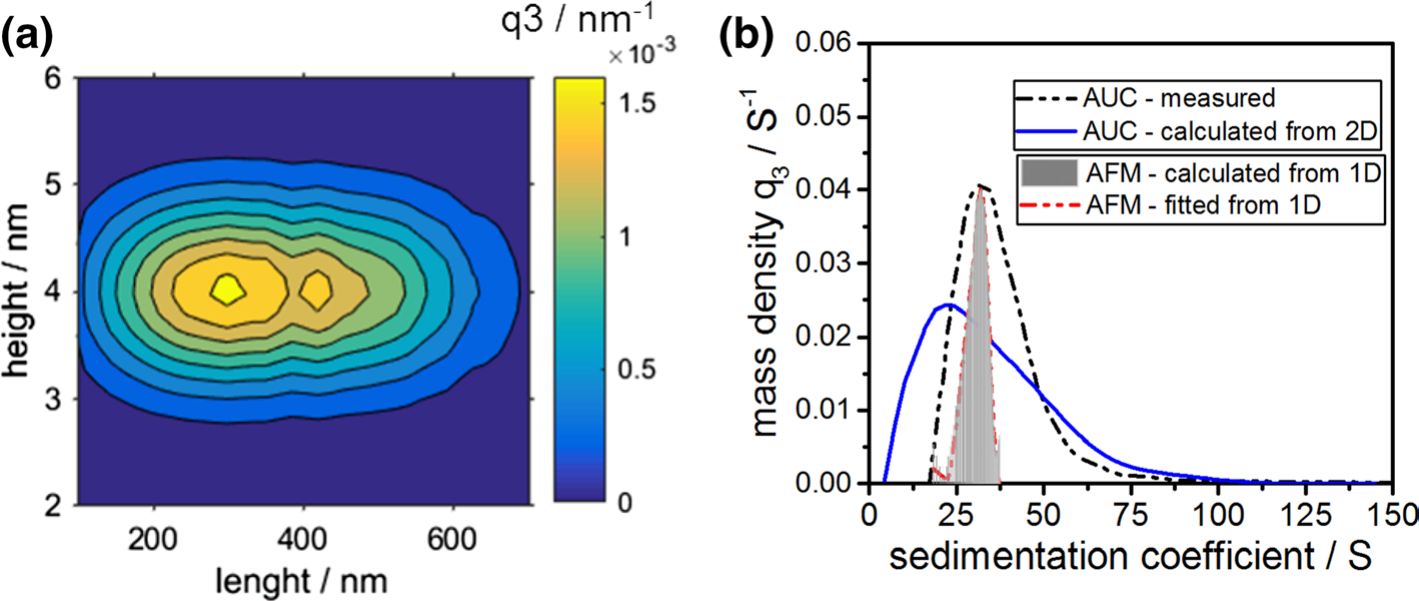
**a** 2D height and length mass-weighted density distribution of BLG fibrils. The underlying length distribution is taken from statistical AFM analysis, while the height distribution is assumed to be normally distributed. The two distributions were considered to be independent when combining them mathematically. **b** Mass density sedimentation coefficient distribution as calculated from a two-dimensional height and length distribution of BLG fibrils (red straight line). Broadening of the sedimentation coefficient distribution is observed with respect to the case of constant fibril height. A mass-weighted sedimentation coefficient distribution from AUC measurement is shown for comparison

A representative length distribution was taken from statistical AFM analysis. Next, a normal height distribution with a mean height of 4.0 nm and a standard deviation of 1 nm was theoretically generated, based on the literature values from Adamcik et al. (2010). These two distributions were then multiplied with each other to obtain a 2D height and length distribution. A schematic illustration of this procedure is provided in the ESI. A representative 2D distribution is displayed in Fig. 8a. For all combinations of length and diameter of the 2D map, sedimentation coefficients are calculated by applying Eq. (4). Eventually, all sedimentation coefficients are classified to retrieve the resulting sedimentation coefficient distributions.

The result is presented in Fig. 8b. For a comparison, the sedimentation coefficient distribution from a one-dimensional length distribution and constant fibril height of 4.7 nm is provided alongside the measured distribution from an AUC experiment.

The width of the one-dimensional sedimentation coefficient distribution increases immensely from ± 5 to ± 35 S when an underlying 2D distribution is assumed. Moreover, the tailing of the distribution at higher sedimentation coefficients (above 50 S) is in good agreement with the results from the auxiliary AUC measurement. Moreover, one can see that the maximum of the sedimentation coefficient distribution calculated from the 2D distribution slightly shifts toward lower sedimentation coefficients compared to the 1D distribution. This is due to the fact that the distribution shows slight tailing at larger sedimentation coefficient, as can be seen in Fig. 8b. The distribution is no longer of Gaussian shape; hence the mean sedimentation coefficient is no longer represented by the modal sedimentation coefficient. However, it is worth mentioning that the mean sedimentation coefficient, calculated from either a 1D or 2D distribution, remains with a good approximation constant and thus in perfect agreement with each other.

At rather low sedimentation coefficients (below 15 S), the results from the AUC measurement do not match the prediction from the 2D distribution. This is attributed to the fact that short fibril lengths in combination with low fibril heights are merely a product of the theoretical considerations and have been observed in neither the statistical AFM analysis nor AUC measurements. Thus, the presence of fibrils exhibiting these dimensions can be excluded and are therefore not present in solution.

In summary, this short theoretical discussion demonstrates well how the increase in the width of the sedimentation properties of BLG fibrils is caused by the fact that both geometrical parameters, namely height and length, are distributed properties.

Notably, the properties measured via AUC include the effect of the entire 2D distribution. Moreover, slight contributions from side chains within the fibril structure, such as H-bonding sites and branched residues (Chou et al. 1983; Wouters and Curmi 1995), affect the orientation and intermolecular interactions within fibrils which might result in a slightly different sedimentation behaviour. However, as these side chains contribute only marginally to the mass of the fibrils, these minor differences in the sedimentation coefficient cannot be resolved using AUC and must therefore be neglected. Furthermore, any effects of concentration-dependent non-ideality are reduced due to the very low concentration (0.01 wt%) (Uttinger et al. 2017). With this, we propose to establish sedimentation properties of amyloid fibrils as a representative parameter to study their functionality and effects.

### Determination of the mean fibril length from the mean and modal sedimentation coefficient as a function of the energy input

**Fig. 9:**
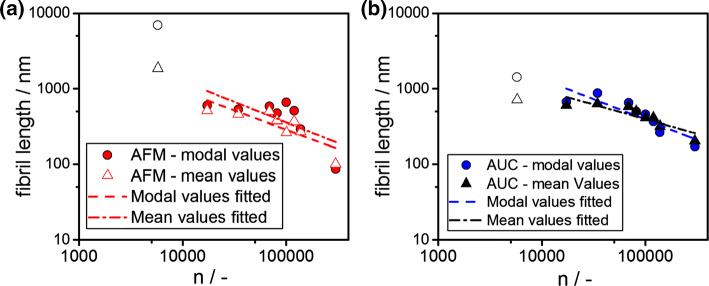
**a** Mean and modal fibril lengths as a function of the stressing conditions as measured by statistical AFM analysis. **b** Calculated fibril length from mean and modal sedimentation coefficient as a function of the stressing conditions as retrieved from AUC measurements

As clearly evident in Fig. 6b, the modal sedimentation coefficient scales with the number of rotations. In the following, insight is provided into the underlying fibril comminution mechanism using Ultra Turrax. Furthermore, the results from both AFM and AUC analysis are compared and differences are discussed in detail. Mean and modal fibril lengths, as measured by statistical AFM analysis, are displayed in Fig. 9a. It is evident that all fibril lengths from AFM measurements as a function of the number of rotations exhibit a linear dependency in the logarithmic plot. The energy input is a function of the rotor geometry as well as the slit geometry at the rotor. Each rotor stator setup has its own characteristics in terms of energy input and performance (Håkansson 2018; Zhang et al. 2012). Here, we applied the number of rotations to evaluate the comminution kinetics of BLG fibrils.

As indicated in Fig. 9a and b, one data point is excluded from the analysis. This data point corresponds to stressing conditions of 11000 rpm for 5 s. Within this short time, the flow pattern around the stirrer is not fully developed. Furthermore, mean and modal fibril lengths are obtained from AUC and the values are displayed in Fig. 9a. The calculations of the fibril lengths from measured sedimentation properties of AUC experiments is carried out based on Eqs. (4) and (5). Again, the effective density is taken from MD simulations and the calculations are performed assuming a constant fibril height of 4.7 nm. The later assumption is applied since it is impossible to retrieve information on the entire 2D distribution from AUC experiments for this system. However, the mean and modal values are discussed, which will provide first insight into the comminution kinetics.

As expected and clearly evident in Fig. 9b, modal fibril lengths taken from AUC experiments and statistical AFM analysis are in the order of 100–800 nm and follow a linear relationship in the logarithmic plot.

For this range, the relative differences from the linear trends are in the order of 5–10%, which is due to the high sample polydispersity and complexity of the fibril structure in solution. Furthermore, small differences may be attributed to a change in flexibility, which can be quantified by the ratio of contour to persistence length (Adamcik und Mezzenga 2012). Further influences might be specific interactions of several fibrils, which lead to coiling and further aggregating effects that in turn affect the sedimentation properties. Hence, even though the ratio of contour to persistence length is in the order of one, a certain degree of flexibility might be maintained (Sagis et al. 2004).

From a combination of Eqs. (8) and (9), one can derive the following relationship between the number of rotations *n* and the applied energy per volume *E*_*V*_:

11 $$E_{V} \sim n^{3}$$

Consequently, considering Eq. (7), the fibril lengths can be related in good approximation to the number of rotations as well as the applied energy per volume E_V_ according to the following relationship:

12 $$l_{{{\text{fibril}}}} \sim E_{v}^{ - b} \sim n^{ - 3b} .$$

**Fig. 10:**
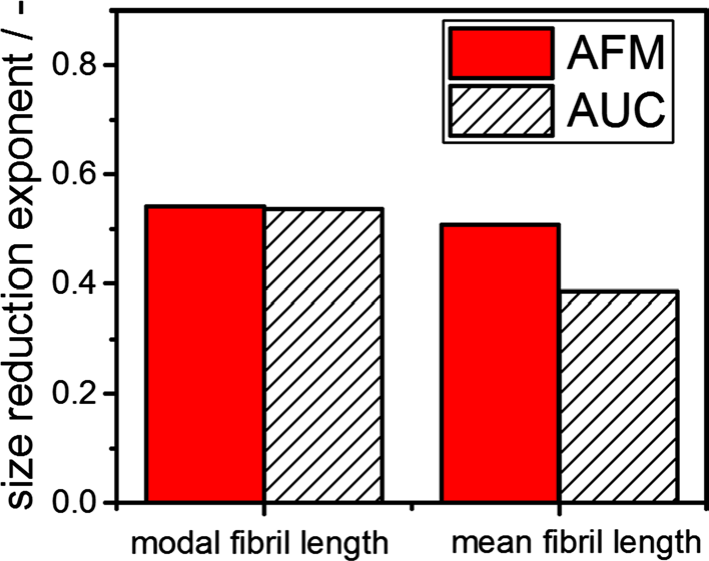
Size reduction exponent b as determined from the mean and modal fibril length. The parameter is retrieved from AFM and AUC measurements

Hence, the size reduction exponent b is determined for mean and modal fibril lengths as indicated by the linear fits in Fig. 9 and the retrieved values are presented in Fig. 10.

Evidently, the size reduction exponents for the modal fibril length agree very well. The differences for the mean values are attributed to the wider distributions of the AUC measurements in comparison with the AFM data. This effect is not constant within each individual sample which accounts for the scatter in the trends in Fig. 9b.

The fibril length scales with the volume specific energy input as shown in the ESI. Given that Ne is only approximately constant under the applied operating conditions of Ultraturrax, the dependency is within the expected range.

Remarkably, the size reduction exponent is in the same order of magnitude as the results for completely different material systems and stressing conditions, namely the size reduction of graphene oxide (Nacken et al. 2017), and other processes such as spraying (Walzel 1990) and emulsification (Behrend et al. 2000). These results underline the importance of fundamental scaling laws for size reduction induced by fluid stresses. In summary, modal sedimentation coefficient as measured from AUC can be applied to study the comminution kinetic of fibrils directly in solution. This enables a fast, robust and reliable analysis of various systems. Hence, the presented method enables the efficient evaluation of fibril properties in dependence of relevant processing parameters directly in solution. We believe that based on our results, the fibril analysis via AUC and the investigation of the size reduction exponent can be extended to other relevant material systems. Hence, future directions on this topic go toward expanding this procedure to amyloids with different building blocks, such as α-synuclein, the tau protein or prion proteins, which play an essential role in neurodegenerative diseases, such as Alzheimer’s or Parkinson disease.

## Summary and conclusion

Amyloid BLG fibril dispersions were stressed and fragmented using an Ultra Turrax. The BLG fibrils were analyzed by statistical AFM analysis and AUC experiments. The measured height and length data from AFM were converted into sedimentation coefficient distributions. For thorough calculations, the relative density changes from the native BLG to the more dense fibrils were obtained from MD simulations. To account for drying effects upon sample preparation for AFM measurements, two surrounding water layers were added to the height of fibrils for the calculation of sedimentation properties in solution. The maxima of both sedimentation coefficient distributions *s*_max_ agree very well.

Furthermore, we theoretically studied the influence of a 2D length and height distribution of the fragmented fibrils and related it to the 1D sedimentation properties under the assumption of a constant fibril height. We propose to utilize the sedimentation coefficient distribution *q*_S_ to describe fibrils in solution, since q_S_ contains information of fibril length, diameter and density.

Material-dependent size reduction parameters retrieved from statistical AFM and AUC measurements match perfectly. The underlying scaling law for the sedimentation coefficient or the equivalent fibril length as a function of the applied stress intensity acting in the Ultra Turrax is very similar to that for other material systems and top-down processes including grinding, emulsification and spraying.

## Electronic supplementary material

Below is the link to the electronic supplementary material.

Supplementary file1 (PDF 445 kb)

## Abbreviations

BLG: Beta-Lactoglobulin
AFM: Atomic force microscopy
AUC: Analytical ultracentrifugation
BD: Brownian dynamics
NAM: Non-aggregated material
MD simulations: Molecular dynamics simulations
ESI: Electronic supporting information
