# Supplementary material for: Measurement of length distribution of beta-lactoglobulin fibrils by multiwavelength analytical ultracentrifugation
Source: Eur Biophys J. 2020 Jan 31;49(8):745–60. doi: 10.1007/s00249-020-01421-4 (PMC7701075; doi:10.1007/s00249-020-01421-4)
Supplement: Supplementary file 1 — Supplementary file1 (PDF 445 kb) [file 249_2020_1421_MOESM1_ESM.pdf]

Electronic supporting information

Measurement of Length Distribution of Beta-Lactoglobulin Fibrils by Multiwavelength Analytical Ultracentrifugation

Maximilian J. Uttinger[1], Timon R. Heyn[2], Uwe Jandt[3], Simon E. Wawra[1], Bettina Winzer[1], Julia K. Keppler[2,4] and Wolfgang Peukert[1*]

[1]Institute of Particle Technology, Interdisciplinary Center for Functional Particle Systems, Friedrich-Alexander-Universität Erlangen-Nürnberg, Erlangen, Germany;

[2]Institute of Human Nutrition and Food Science, Division of Food Technology, Kiel University, 24118 Kiel, Germany

[3]Institute of Bioprocess and Biosystems Engineering Hamburg University of Technology, Germany

[4]Laboratory of Food Process Engineering, Wageningen University, Bornse Weilanden 9, 6708WG, Wageningen, P.O. Box 17, 6700 AA, Wageningen, the Netherlands

*corresponding author

Contact

Maximilian J. Uttinger: max.uttinger@fau.de

Timon R. Heyn: theyn@foodtech.uni-kiel.de

Simon E. Wawra: simon.wawra@fau.de

Bettina Winzer: Bettina.winzer@fau.de

Julia K. Keppler: julia.keppler@wur.nl

*Wolfgang Peukert: wolfgang.peukert@fau.de

Assessment of effective fibril densities using molecular dynamics

The systems were energy minimized using the steepest descent algorithm(Cauchy 1847) to eliminate any overlap or clash between the atoms. Then, it was equilibrated under an NVT ensemble for 20 ps and a single integration time step of 2 fs with position restraints on the heavy atoms of the protein leading to a relaxation of the surrounding solvent molecules. The temperature was maintained at the reference value of 293 K using V-rescale (modified Berendsen thermostat) with a coupling time constant of TC = 0.1 ps. The pressure was kept at 1 bar using a Parrinello-Rahman barostat with a coupling constant of 2 ps. The isothermal compressibility value used for the pressure coupling algorithm for water was 4.5 10-5 bar-1. The long range interactions were applied using the Particle Mesh Ewald (PME) method with a real space cut-off of 1 nm, a Fourier mesh spacing of 0.16 nm and fourth-order interpolation. The Lennard-Jones interactions were calculated using a cut-off of 1.0 nm. After the systems were well equilibrated, short production MD runs were performed for a simulated time of t = 2 ns until the root mean square deviation (RMSD) ceased. This allowed minor structural adaptations and possible opening or closure of small pockets. The finally obtained simulation boxes for native □BLG and □beta-sheet amyloids were then subjected to further analysis.

Mass and density distribution

$$q_0(l) = \frac{number\ in\ l + \Delta l}{Total\ number\ of\ fibrils \cdot \Delta l}$$

$$q_3(l) = \frac{mass\ in\ l + \Delta l}{Total\ mass\ of\ fibrils \cdot \Delta l}$$

List of stressed samples

Sample Nr / #	Rotational speed / rpm	Time / s	ω t /-	n / -	E_V / J mL^{-1}
1	11000	30	34557.52	5500	6.49
2	19000	60	119380.52	19000	534.79
3	24000	120	301592.89	48000	17245.44
4	11000	5	5759.59	916.67	0.01
5	11000	15	17278.76	2750	0.41
6	11000	60	69115.038	11000	103.78
7	13000	60	81681.41	13000	171.30
8	16000	60	100530.96	16000	319.36
9	22000	60	138230.08	22000	830.21

Calculations were performed based on Equations 8 to 10 of the manusript.

Statistical AFM image analysis

The results from statistical AFM analysis for different stressing conditions is provided.

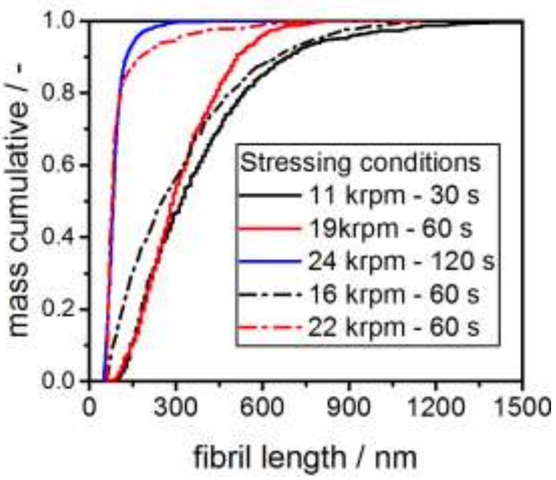

Figure S1: Mass cumulative distribution of the fibril length from analyzing the AFM image.

Dynamic Light Scattering – DLS

Stressed samples were diluted to 1 mg/ml and measured in a Zetasizer Nano ZS (*Malvern Instruments GmbH, Herrenberg, Germany*) using disposable cuvettes DTS0012. Temperature during measurement was set to 25°C. A viscosity of 0.8872 cP and a material refractive index of 1.450 were used.

DLS and AFM- mass weighted length distributions

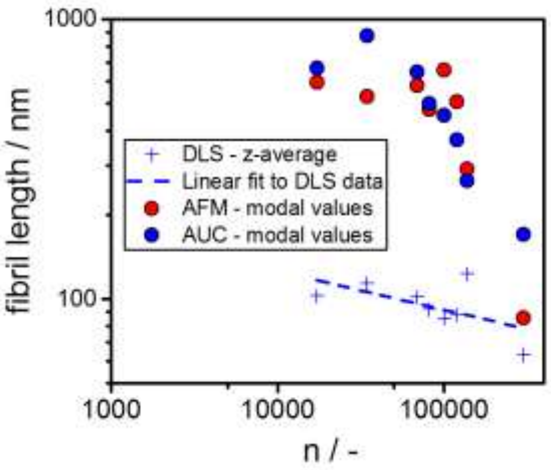

Figure S2: Mean fibril length as a function of the stressing condition from AUC measurements. For comparison, the z-average from DLS measurements is depicted as a function of the stressing condition.

Length reduction kinetic

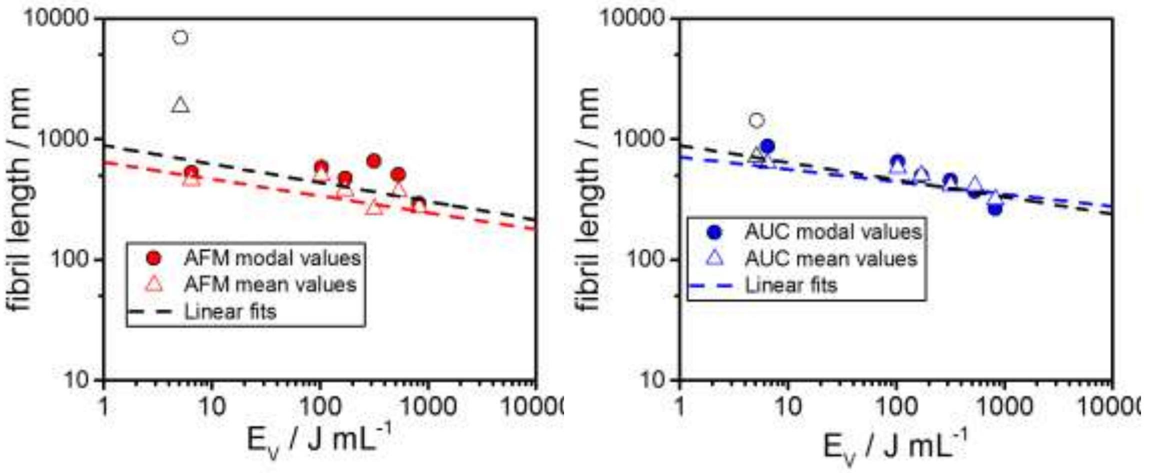

Figure S3: Left: Mean and modal fibril lengths as a function of the stressing conditions as measured by statistical AFM analysis. Right: Calculated fibril length from mean and modal sedimentation coefficient as a function of the stressing conditions as retrieved from AUC measurements.

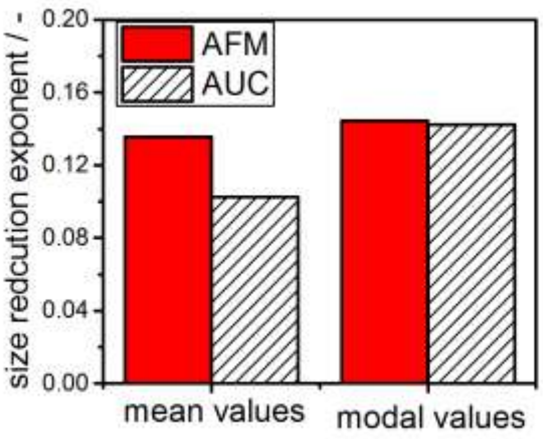

Figure S4: Size reduction exponent as determined from the mean and modal fibril length. The parameter is retrieved from AFM and AUC measurements.
